# An Improved Method for Dynamic Measurement of Deflections of the Vertical Based on the Maintenance of Attitude Reference

**DOI:** 10.3390/s140916322

**Published:** 2014-09-03

**Authors:** Dongkai Dai, Xingshu Wang, Dejun Zhan, Zongsheng Huang

**Affiliations:** College of Opto-Electronic Science and Engineering, National University of Defense Technology, Deya Street 109, Changsha 410073, China; E-Mails: gfkdwxs@gmail.com (X.W.); zdj4444@sina.com (D.Z.); zongsheng_huang@sina.com (Z.H.)

**Keywords:** deflections of the vertical, INS/GNSS integration, attitude reference, EGM2008

## Abstract

A new method for dynamic measurement of deflections of the vertical (DOV) is proposed in this paper. The integration of an inertial navigation system (INS) and global navigation satellite system (GNSS) is constructed to measure the body's attitude with respect to the astronomical coordinates. Simultaneously, the attitude with respect to the geodetic coordinates is initially measured by a star sensor under quasi-static condition and then maintained by the laser gyroscope unit (LGU), which is composed of three gyroscopes in the INS, when the vehicle travels along survey lines. Deflections of the vertical are calculated by using the difference between the attitudes with respect to the geodetic coordinates and astronomical coordinates. Moreover, an algorithm for removing the trend error of the vertical deflections is developed with the aid of Earth Gravitational Model 2008 (EGM2008). In comparison with traditional methods, the new method required less accurate GNSS, because the dynamic acceleration calculation is avoided. The errors of inertial sensors are well resolved in the INS/GNSS integration, which is implemented by a Rauch–Tung–Striebel (RTS) smoother. In addition, a single-axis indexed INS is adopted to improve the observability of the system errors and to restrain the inertial sensor errors. The proposed method is validated by Monte Carlo simulations. The results show that deflections of the vertical can achieve a precision of better than 1″ for a single survey line. The proposed method can be applied to a gravimetry system based on a ground vehicle or ship with a speed lower than 25 m/s.

## 1. Introduction

High resolution and high precision gravity data are desirable in many applications, such as geodesy, solid earth geophysics and resource exploration. Although the global gravity disturbances can be measured effectively by satellite gravimetry, the detailed information of the gravity field is poorly determined [1]. Therefore, the moving-based gravimetry system, based either on an aircraft, ground vehicle or ship, serves as a good alternative for the gravity disturbance measurement in the frequency range from medium to high. Currently, the vertical component of the gravity disturbance vector is successfully measured by sea/air-borne scalar gravimetry [2–4]; however, the horizontal components are still difficult to measure under dynamic conditions.

The horizontal components (with respect to the ellipsoid) of the gravity disturbance vector, can be determined by deflections of the vertical (DOV), which are known as the direction difference between the actual plumb line and the normal of the ellipsoid. The astronomic-geodetic observation with instrumentation, such as theodolites and digital zenith cameras [5], is a high-precision and traditional method to measure deflections of the vertical. Since the astronomic observation must be conducted on the ground statically, the method is less efficient than moving-based gravimetry. There are also other methods capable of providing DOV, such as the combination of GNSS and leveling measurements [6], the combination of GNSS and a local positioning system (LPS) [7], *etc.*; however, these methods have relatively poor accuracy.

In comparison with the scalar gravimetry, the vector gravimetry based on the GNSS/INS integration is more challenging, and it can measure all three components of gravity disturbances under dynamic conditions. Two main methods for vector gravimetry were developed in the last four decades.

The first method was developed by Rose and Nash [8]. In their research, the gravity disturbances were modeled as second-order Gauss–Markov processes [9,10] and directly estimated by a Kalman filter/smoother using an inertial navigation system with the aid of external position and velocity updates. A series of studies were subsequently carried out by a number of researchers to improve this method. These studies mainly focus on gravity disturbance modeling. According to Jekeli's study [11], it is expected that the short-wavelength gravity components could be estimated in the presence of the stable uncompensated gyro drift when the gravity disturbances were modeled as low-order Gauss–Markov processes. However, this method is not adopted by most of the actual systems, because it is difficult to model the gravity disturbances accurately.

To overcome the short-coming of the method mentioned above, Kwon and Jekeli [12] proposed a model-free approach for gravity vector measurement. In their study, the gravity disturbances are calculated by the difference between the GNSS-derived accelerations and INS-sensed specific force in the absence of any gravity stochastic models. This kind of method has been greatly improved and widely adopted by many vector gravimetry systems in the last two decades. When several types of error separation methods, such as wavenumber correlation filter (WCF) [13] and wavelet de-noising techniques [14], were applied, promising results were obtained. A typical system based on this method was developed by Ohio State University in 2006. The experiment results showed that the extent of accuracy of this system can reach 7–8 mGal (1 mGal = 1e−5 m/s^2^) for horizontal components. The renewed airborne gravimetry of Sander Geophysics Ltd., namely AIRGrav (Airborne Gravimetry), is another system of this kind. The internal accuracy of the system can reach 0.5 mGal for horizontal components, as reported [15]. Although the model-free method can achieve high precision, there are still several disadvantages. First, The WCF technique, which is adopted by most systems, demands repeated track data for the surveys. Consequently, the cost and time requirements are burdensome. Second, the differential GNSS technology for calculating the kinematic accelerations is indispensable. Because the acceleration computation is implemented by a second-order differentiator [16], which is essentially a high pass filter, the GNSS noise will be enhanced. Additionally, it is necessary to establish GNSS reference stations around the survey routes for differential GNSS; however, the reference stations are difficult to setup in some regions.

In this paper, we propose a new approach for dynamic measurement of DOV. An INS/GNSS integration is constructed to measure the body's attitude with respect to the astronomical coordinates. Simultaneously, the attitude with respect to the geodetic coordinates is initially measured by a star sensor and then maintained by three laser gyroscopes in INS, namely the laser gyroscope unit (LGU). DOVs are calculated by using the difference between the attitudes with respect to the geodetic coordinates and astronomical coordinates. Finally, the systematic errors are reduced with the aid of the global gravity model.

The new method has three features, which are different from traditional vector gravimetry. First, an independent attitude reference is provided by the star sensor and LGU. Hence, the error of the attitude reference is not coupled with DOV. Second, the calculation of the kinetic acceleration is avoided in the new method, and thus, it requires less accurate GNSS measurement. Third, a single-axis indexed inertial navigation system is adopted to enhance the observability of the system errors and restrain the bias errors of the inertial sensors. Because of these three features, the errors of attitude reference, GNSS and INS will be well handled, respectively.

The remainder of this paper is organized as follows. Section 2 will introduce the theoretical development of the new method for DOV measurement in detail. The simulation procedure and the preliminary results are presented in Section 3. The viability of the new method based on the detailed error analysis will be discussed in Section 4; the error correction algorithm will also be proposed. Finally, the conclusions will be given in Section 5.

## 2. The Method for DOV Measurement

**Figure 1.**
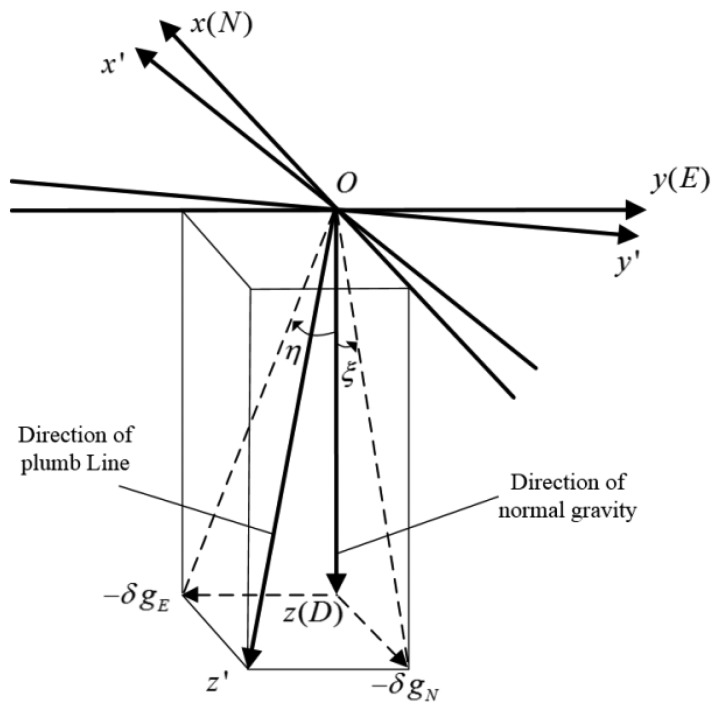
The definition of coordinates and deflections of the vertical (DOVs).

Initially, we define two kinds of coordinate frames, namely the astronomical coordinates and the geodetic coordinates. As Figure 1 shows, the geodetic coordinates *O*-*xyz* (*n*-frame) are relative to the normal ellipsoid. The origin *O*-point is at the measurement site. The *z*-axis points toward the interior of the ellipsoid along the ellipsoid normal (*D*), which is in the direction of the normal gravity vector through *O*. The *x*-axis points toward north (*N*), and the *y*-axis points east (*E*) to complete the orthogonal, right-handed rectangular coordinate system. The astronomical coordinates *O*-*x′y′z′*(*n′*-frame), which are related to the actual geoid, are defined as follows: The *z′*-axis points toward the interior of the geoid along the geoid normal, which is in the direction of the actual gravity vector through *O*. The *x′*-axis points toward north, and the *y′*-axis points east to complete the orthogonal, right-handed rectangular coordinate system.

As shown in Figure 1, the north and east components of the gravity disturbance vector are denoted by *δg_N_* (the positive direction is toward north) and *δg_E_* (the positive direction is toward east), respectively. The north-south and east-west angular components of DOV are denoted by *ξ* and *η*, which are directly related to the horizontal components of the gravity disturbance vector and defined by:

(1) $$\tan\xi =-\delta g_{N}/g$$

(2) $$\tan\eta =-\delta g_{E}/g$$

where *g* is the magnitude of the normal gravity. For small angles, Equations (1) and (2) can be approximately written as:

(3) $$\xi \approx -\delta g_{N}/g$$

(4) $$\eta \approx -\delta g_{E}/g$$

Since the *n*-frame and *n′*-frame are defined by the physical plumb line and the normal gravity, the signature of DOV is reflected in the coordinate transformation matrix from *n*-frame to *n′*-frame $C_{n}^{n'}$. $C_{n}^{n'}$ is determined by Equation (5) with DOVs [17]. In essence, the key problem with DOV measurement is to obtain the transformation matrix $C_{n}^{n'}$.

(5) $$C_{n}^{n'}\approx \left[\begin{array}{ccc} 1 & -\delta \alpha & \xi \\ \delta \alpha & 1 & \eta \\ -\xi & -\eta & 1 \end{array}\right]$$

where *δα* represents the final rotation around the astronomical zenith axis to make the geodetic coordinates and astronomical coordinates coincident. According to [17], *δα* = *η* tan *L*, where *L* is the local latitude.

### 2.1. INS/GNSS Integrated System for Geoid Tracking

The system error model of INS can be expressed by a set of linear, first-order, differential equations [18]. The dynamics of the velocity error defined in *n*-frame are given by:

(6) $$\delta \dot{V}=f^{n}\times \Phi -(2\delta \omega_{ie}^{n}+\delta \omega_{en}^{n})\times V-(2\omega_{ie}^{n}+\omega_{en}^{n})\times \delta V+C_{b}^{n}\nabla^{b}+\delta g^{n}$$

where **V** is the velocity of INS and *δ***V** is the velocity error of INS; **f***^n^* is the specific force in *n*-frame; **Φ** is the attitude error of INS; *δ***g***^n^* is the gravity disturbance vector in *n*-frame, which is the error of gravity information in the knowledge of normal gravity, and given by *δ***g***^n^* = **g***^n^* − **γ***^n^*, where **g***^n^* and **γ***^n^* are the actual gravity vector and normal gravity vector in the *n*-frame, respectively; $\omega_{ie}^{n}$ is the Earth's rotation rate with respect to the inertial reference frame in *n*-frame; $\omega_{en}^{n}$ is the rotation rate of the *n*-frame with respect to Earth-fixed frame (*e*-frame) in *n*-frame; $\delta \omega_{ie}^{n}$ and $\delta \omega_{en}^{n}$ are the errors of $\omega_{ie}^{n}$ and $\omega_{en}^{n}$, which are in terms of the position and velocity error of INS, respectively; ∇*^b^* is the accelerometer bias in the body frame (*b*-frame); $C_{b}^{n}$ is the transformation matrix from the *b*-frame to the *n*-frame.

The dynamic equation of the attitude error based on the phi-angle error model [19] is given by:

(7) $$\dot{\Phi }=-(\omega_{ie}^{n}+\omega_{en}^{n})\times \Phi +\delta \omega_{ie}^{n}+\delta \omega_{en}^{n}-C_{b}^{n}\varepsilon^{b}$$

where **ε***^b^* is the gyroscope bias in the *b*-frame.

The differential equation of the position error is given as follows:

(8) $$\delta \dot{P}=\delta V$$

The biases of the accelerometer and gyroscope can be modeled as random constants, which are respectively given by:

(9) $${\dot{\nabla }}^{b}=0$$

(10) $${\dot{\varepsilon }}^{b}=0$$

Equations (6)–(10) can be rewritten together succinctly as:

(11) $$\delta \dot{x}=F\cdot \delta x+Gw$$

where the state-space vector is given by Equation (12); **F** is the state transition matrix, which is readily constructed from Equations (6)–(10). It should be noted that the gravity disturbances *δ***g***^n^* are ignored in the state-space equation. The gravity-induced attitude errors will be discussed later.

(12) $$\delta x={\left[\delta V^{T},\Phi^{T},\delta P^{T},{\left[\nabla^{b}\right]}^{T},{\left[\varepsilon^{b}\right]}^{T}\right]}^{T}$$

The process noise **w** = [[ **w***_g_*]*^T^*, [**w***_a_*]*^T^*]*^T^* is composed of the intensity of gyroscope Gaussian white-noise **w***_g_* and accelerometer Gaussian white-noise **w***_a_*. The model error distribution matrix **G** is given by:

(13) $$G=\left[\begin{array}{cc} O_{3\times 3} & C_{b}^{n}\\ C_{b}^{n} & O_{3\times 3}\\ O_{9\times 3} & O_{9\times 3} \end{array}\right]$$

As the GNSS can provide position and Doppler velocity information, the velocity error and position error of INS can be observed directly and chosen as measurements. The measurement vector is defined as **z** = [*δ***V***^T^*, *δ***P***^T^*]*^T^*. The measurement model is expressed as:

(14) z=Hδx+v

where $H=\left[\begin{array}{cccc} I_{3\times 3} & O_{3\times 3} & O_{3\times 3} & O_{3\times 6}\\ O_{3\times 3} & O_{3\times 3} & I_{3\times 3} & O_{3\times 6} \end{array}\right]$, **v** is the GNSS position and velocity observation white-noise error.

The underlying estimation algorithm for integrated navigation is the Kalman filter [20]. The mission objective has no demand for real-time processing; therefore, a fixed-interval smoother would be more appropriate. A Rauch–Tung–Striebel (RTS) smoother algorithm [21] is adopted in this paper.

Next, we will investigate the effect of the gravity disturbances on the attitude output of INS/GNSS integration by using error-free inertial sensors (the gravity disturbances are the only error source). Equation (6) can be simplified as Equation (15) when the term $C_{b}^{n}\nabla^{b}$ is ignored.

(15) $$\delta \dot{V}=f^{n}\times \Phi +\delta g^{n}-(2\delta \omega_{ie}^{n}+\delta \omega_{en}^{n})\times V-(2\omega_{ie}^{n}+\omega_{en}^{n})\times \delta V$$

When the INS is aided with GNSS, which can provide accurate position and velocity updates, the variables *δ***V**, $\omega_{ie}^{n}$, $\omega_{en}^{n}$, $\delta \omega_{ie}^{n}$, $\delta \omega_{en}^{n}$ and **V** can be observed directly. Equation (11) can be rewritten as:

(16) $$y=f^{n}\times \Phi +\delta g^{n}$$

where $y=\delta \dot{V}+(2\delta \omega_{ie}^{n}+\delta \omega_{en}^{n})\times V+(2\omega_{ie}^{n}+\omega_{en}^{n})\times \delta V$, which is the combination of observable variables.

The north and east components of Equation (15) can be respectively described as:

(17) $$y_{N}=-f_{E}\phi_{D}+f_{D}\phi_{E}+\delta g_{N}$$

(18) $$y_{E}=-f_{D}\phi_{N}+f_{N}\phi_{D}+\delta g_{E}$$

where *y_N_* and *y_E_* are the north and east components of **y**; *ϕ_N_*, *ϕ_E_* and *ϕ_D_* are the north, east and down components of **Φ**; *f_N_*, *f_E_* and *f_D_* are the north, east and down components of **f***^n^*.

Assuming that the vehicle is cruising with constant velocity, which is met under most conditions when the survey is conducted, we have *f_E_ϕ_D_* ≈ 0, *f_N_ϕ_D_* ≈ 0 and *f_D_* ≈ *g*. Equations (17) and (18) can be rewritten as follows:

(19) $$\phi_{E}=y_{N}/g-\delta g_{N}/g$$

(20) $$\phi_{N}=\delta g_{E}/g-y_{E}/g$$

The observation components of *ϕ_E_* and *ϕ_N_* are defined as *ϕ̂_E_* = *y_N_*/*g* and *ϕ̂_N_* = −*y_E_*/*g*, respectively. Substituting Equations (3) and (4) into Equations (19) and (20), we have:

(21) $${\hat{\phi }}_{E}=\phi_{E}+\xi$$

(22) $${\hat{\phi }}_{N}=\phi_{N}+\eta$$

The observation errors of *ϕ_E_* and *ϕ_N_* are defined as *δϕ_E_* = *ϕ̂_E_* − *ϕ_E_* and *δϕ_N_* = *ϕ̂_N_* − *ϕ_N_*, which are the attitude measurement errors of INS/GNSS integration. According to Equations (21) and (22), we have:

(23) $$\delta \phi_{E}=-\xi$$

(24) $$\delta \phi_{N}=\eta$$

In other words, the signatures of DOV can be reflected in the attitude measurement errors of INS/GNSS integration.

The attitude error matrix of INS/GNSS is given by Equation (25) [18].

(25) $$E=\left[\begin{array}{ccc} 0 & -\delta \phi_{D} & \delta \phi_{E}\\ \delta \phi_{D} & 0 & -\delta \phi_{N}\\ -\delta \phi_{E} & \delta \phi_{N} & 0 \end{array}\right]$$

where *δϕ_D_* is the observation error of *ϕ_D_*. The transformation matrix from the true reference frame to the estimated reference is given by **I** − **E**, where **I** is a 3 × 3 identify matrix. Substituting for *δϕ_E_* and *δϕ_N_* with Equations (23) and (24),

(26) $$I-E=\left[\begin{array}{ccc} 1 & \delta \phi_{D} & \xi \\ -\delta \phi_{D} & 1 & \eta \\ -\xi & -\eta & 1 \end{array}\right]$$

Given that the ideal attitude output of the INS/GNSS integration is $C_{b}^{n}$, the actual attitude output of the INS/GNSS system is $(I-E)C_{b}^{n}$. It is easy to find that $I-E\approx C_{n}^{n'}$ when ignoring the small angular difference between *δϕ_D_* and *δα*. It is indicated that the actual attitude output approximately equals $C_{b}^{n'}$. Therefore, the INS/GNSS integration measures the body's attitude with respect to the *n′*-frame.

### 2.2. An LGU to Maintain the Attitude Reference

**Figure 2.**
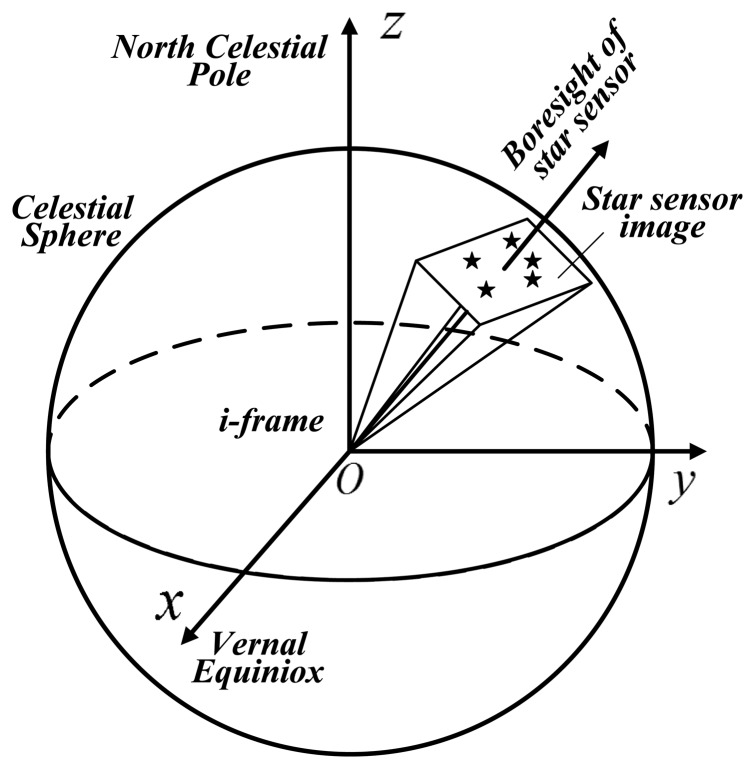
The principal of the star sensor.

As shown in Figure 2, a star sensor [22] can be applied to determine the attitude with respect to the celestial coordinates, which is an inertial frame system (*i*-frame), by astronomical observation. The inertial frame can be transformed to the Earth-fixed frame using the Greenwich Apparent Sidereal Time (GAST). When the position of the measurement site is available, the attitude matrix $C_{b}^{n}$ with respect to the *n*-frame can be obtained. The accuracy of the star sensor is typically better than 3″ [23] under static or quasi-static conditions, and a multiple heads star sensor or zenith camera will enhance the performance. In this paper, the star sensor acts as the attitude reference to determine the attitude error (with respect to the *n*-frame) of the INS/GNSS integration.

It should be noted that the attitude of the star sensor is determined by astronomical observation; therefore, the DOV measurement must be carried out in the nighttime with good weather conditions. Moreover, the star sensor might have poor accuracy under dynamic conditions, which limits its application. In order to overcome these disadvantages of the star sensor, we propose to use LGU to maintain the attitude reference obtained by the star sensor. The LGU is composed of three laser gyroscopes in the INS. Thus, no additional gyroscope is need. The laser gyroscopes can measure the rotation motion with respect to the inertial frame with a precision of better than 1″ per sample interval (typically, 0.01 s).

The attitude matrix $C_{b}^{e}$ of LGU with respect to the Earth-fixed frame is initialized by a star sensor under quasi-static conditions in the nighttime. When the measurement starts and the vehicle travels along the survey line dynamically, the LGU can autonomously maintain the attitude reference obtained by star sensor. The attitude of LGU can be updated by the Equation (27).

(27) $$C_{b_{k+1}}^{e_{k+1}}=C_{e_{k}}^{e_{k+1}}C_{b_{k}}^{e_{k}}C_{b_{k+1}}^{b_{k}}$$

where $C_{e_{k}}^{e_{k+1}}$ describes the change of the *e*-frame from time *t_k_* to *t_k_*_+1_; this can be calculated by using GAST. $C_{b_{k+1}}^{b_{k}}$ describes the change of the *b*-frame from *t_k_*_+1_ to *t_k_*, which can be calculated by using the angular increments of the gyroscopes. The attitude with respect to the *n*-frame can be obtained with the aid of GNSS position measurement, which is written as:

(28) $$C_{e}^{n}=\left[\begin{array}{ccc} -\sin\lambda & \cos\lambda & 0\\ -\sin L\cos\lambda & -\sin L\sin\lambda & \cos L\\ \cos L\cos\lambda & \cos L\sin\lambda & \sin L \end{array}\right]$$

(29) $$C_{b}^{n}=C_{e}^{n}C_{b}^{e}$$

where *L* and *λ* are the latitude and longitude of the measurement site, respectively.

$C_{n}^{n'}$ can be determined by the attitude output of INS/GNSS and LGU.

(30) $$C_{n}^{n'}=C_{b}^{n'}{\left(C_{b}^{n}\right)}^{T}$$

Finally, DOVs can be calculated according to Equation (5).

It should be noted that the gyroscope measurements are used in two procedures, firstly in INS/GNSS integration and, secondly, in LGU. According to Equations (23) and (24), we can see that the attitude errors of INS/GNSS integration are determined by DOV, which is introduced into INS/GNSS attitude measurement by accelerometers. While the attitude references provided by LGU are calculated by only gyroscope measurements, so that the attitude reference will not couple with DOV, then, the attitude errors of INS/GNSS integration can be measured.

## 3. Simulation Result

### 3.1. Design of Simulation

In this section, simulations are implemented to investigate the viability of the proposed method. The inertial measurement unit (IMU) is assumed to be of navigation-grade, and only the bias error and white noise of the inertial sensor are considered. The error characteristics of the inertial sensor, GNSS and star sensor are listed in Table 1.

In order to remove most system errors arising from the drifts of the inertial sensors, an INS of a single axis rotation tuning structure is adopted [24]. In the INS/GNSS integrated system, the observability of the system errors can be improved greatly by the rotation tuning. Furthermore, the errors of the inertial sensors will be restrained.

The simulation is carried out with the initializations as follows:

(1) The sample interval of the inertial sensor is 0.05 s, and the update period of GNSS is 1 s in the simulation.
(2) The initial latitude is 35°; the longitude is 240° and the height is 100 m.
(3) Suppose the vehicle remains static for 5 h at the initial time, then accelerates to 20 m/s with an east acceleration of 0.1 m/s^2^ and, finally, travels with a constant speed of 20 m/s heading east at a constant altitude.
(4) Before the survey is carried out, a fine alignment procedure for the INS/GNSS integrated system is executed so that the system can achieve a satisfactory performance.

**Figure 3.**
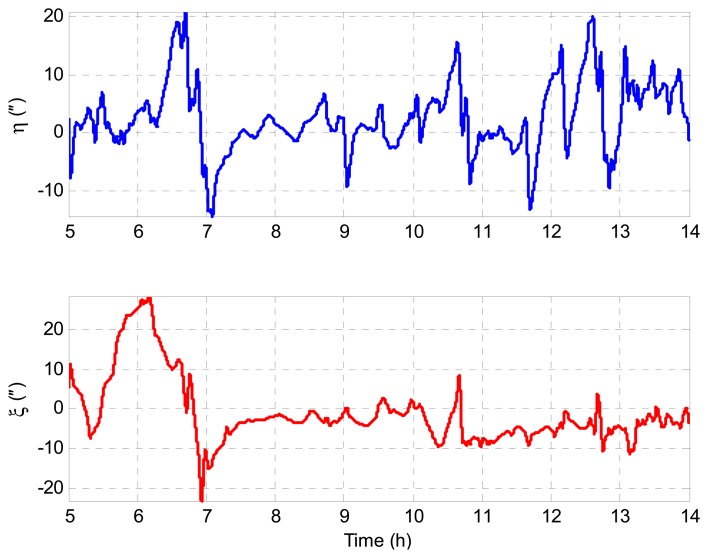
Gravity disturbance distribution along-track.

The gravity disturbances used to generate the IMU data are obtained from a high-precision and high-resolution regional vertical deflection model of the USA, namely DEFLEC12, which is more accurate than the global gravity model. Figure 3 shows the gravity disturbances along the survey line.

### 3.2. Data Processing and Preliminary Result

**Figure 4.**
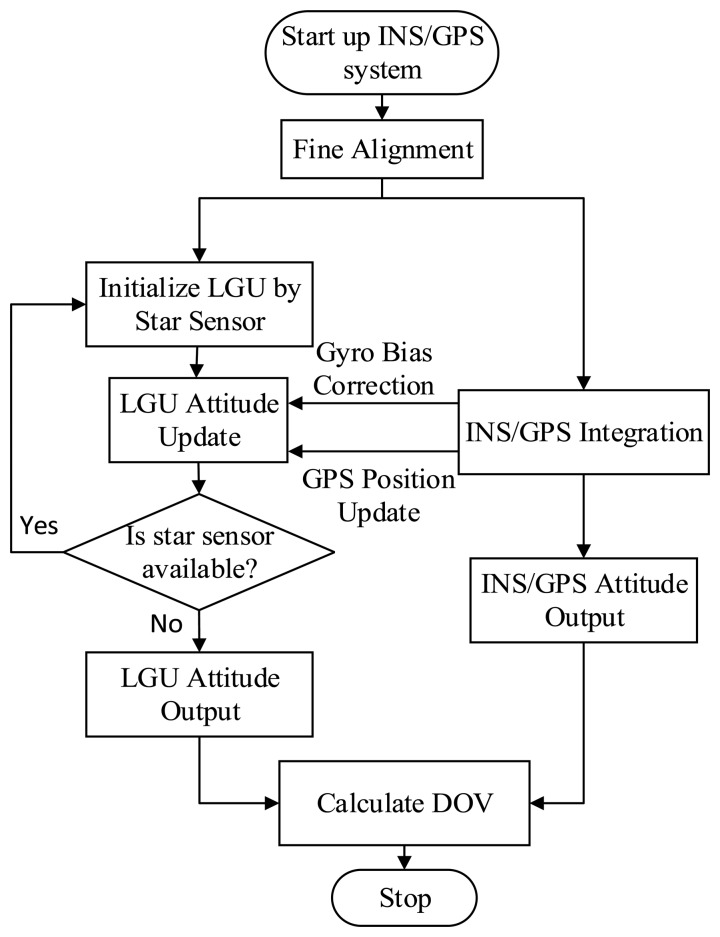
The procedure for DOV measurement. LGU, laser gyroscope unit.

The new method for the vertical deflection determination consists of three main procedures. Firstly, the INS/GNSS integrated system is utilized to measure the attitude with respect to the astronomical coordinates frame (*n′*-frame). An RTS smoother algorithm is adopted to fuse the data of INS and GNSS. Secondly, a star sensor is utilized to measure the attitude with respect to the geodetic coordinates frame (*n*-frame), and the raw gyroscope data are integrated to maintain the attitude reference obtained by the star sensor. The gyroscope biases estimated by the INS/GNSS integrated system are used to correct the raw gyroscope angular increments. Finally, DOVs are calculated by using the $C_{b}^{n'}$ measured by INS/GNSS and $C_{b}^{n}$ measured by LGU. The attitudes of LGU are re-initialized by the star sensor for a period of 4 h when the star sensor's output is available. The procedures of the proposed method are shown in Figure 4.

The viability of the proposed method depends on two assumptions: firstly, the computed platform [19] of INS/GNSS integration is able to track the actual geoid; secondly, the LGU can maintain the attitude reference obtained by the star sensor. Simulations are implemented to verify these two assumptions, and the preliminary results of DOV estimation are also presented in this section.

**Figure 5.**
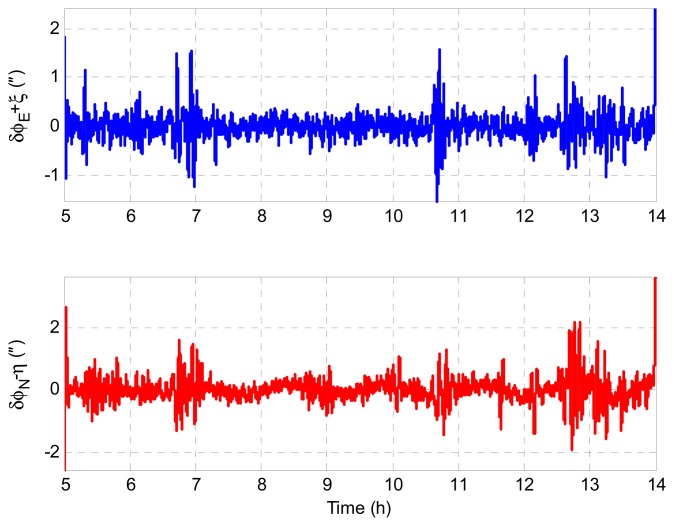
Geoid tracking error for INS/GNSS integration.

**Figure 6.**
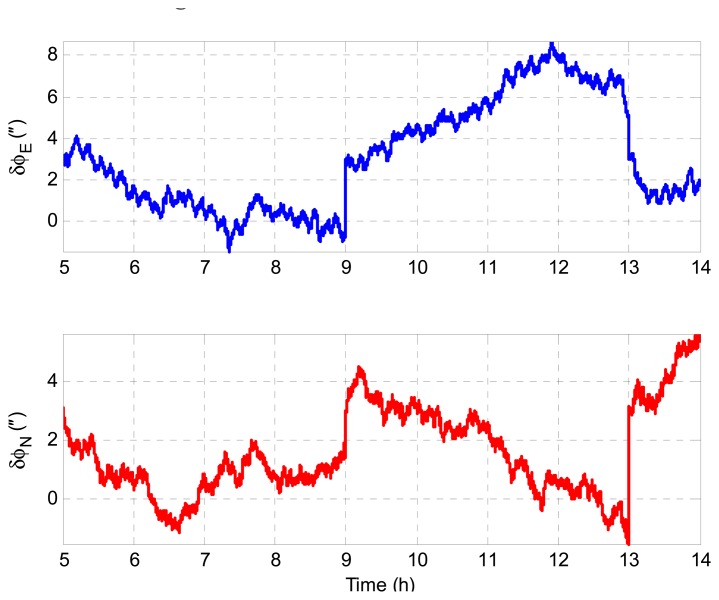
Attitude errors of the LGU.

According to Equations (23) and (24), we have *δϕ_E_* + *ξ* = 0 and *δϕ_N_* − *η* = 0 theoretically. Therefore, the geoid tracking error of INS/GNSS integration can be assessed by *δϕ_E_* + *ξ* and *δϕ_N_* − *η* in simulation. Figure 5 shows the geoid tracking error of INS/GNSS integration. As can be seen, the geoid tracking errors of INS/GNSS integration are near zero, which is consistent with the theory in Equations (23) and (24). It is implied that the DOV signatures are reflected in *δϕ_E_* and *δϕ_N_*. Thus, the key problem of DOV determination is to measure the attitude errors of INS/GNSS. The LGU attitude error with respect to the *n*-frame is shown in Figure 6. It can be seen that the LGU attitude outputs contain considerable systematic trend error, which will introduce significant error into the ultimate DOV measurement results. The attitude error of LGU is mainly caused by the error of gyroscopes and the initialization attitude error. The LGU attitude is initialized by the star sensor. Because the error of the star sensor is considerable, the re-initialization of LGU attitude will introduce some gap errors, which can be seen in Figure 6.

**Figure 7.**
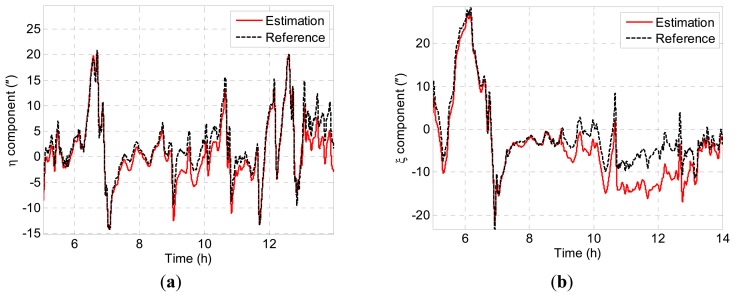
(**a**) Estimation result of *η*; (**b**) estimation result of *ξ*.

Figure 7a,b depicts the preliminary estimation results of DOV. It can be seen that the estimation results contain considerable systematic errors. The statistical analysis of the simulation result is shown in Table 2, where $\delta \phi_{E}^{\text{INS}}$ and $\delta \phi_{N}^{\text{INS}}$ are the east and north components of the attitude error (with respect to the *n*-frame) in INS/GNSS integration; $\delta \phi_{E}^{\text{LGU}}$ and $\delta \phi_{N}^{\text{LGU}}$ are the east and north components of the attitude error (with respect to the *n*-frame) in LGU; the reference values of DOV are given by DELFEC12 model and denoted by *ξ^DEFLEC^*^12^ and *η^DEFLEC^*^12^; and the estimation values of DOV are denoted by *ξ^Estimation^* and *η^Estimation^*. It can be seen from Table 2 that the geoid tracking errors of INS/GNSS are less than 0.5″; the LGU attitude errors, which are relatively larger, can reach 2.62″ and 1.51″ for the east and north components; the DOV estimation errors, which are mainly caused by the LGU attitude errors, can reach 2.62″ and 1.51″ accordingly.

## 4. Error Analysis and Correction

The detailed error budget and correction method will be discussed in this section. The variables of the error analysis include accelerometer accuracy, GNSS accuracy, gyroscope accuracy and travel speed of the vehicle.

### 4.1. Analysis of Geoid Tracking Error

**Figure 8.**
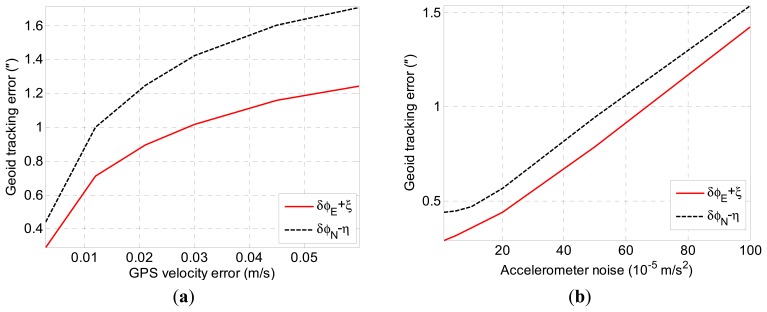

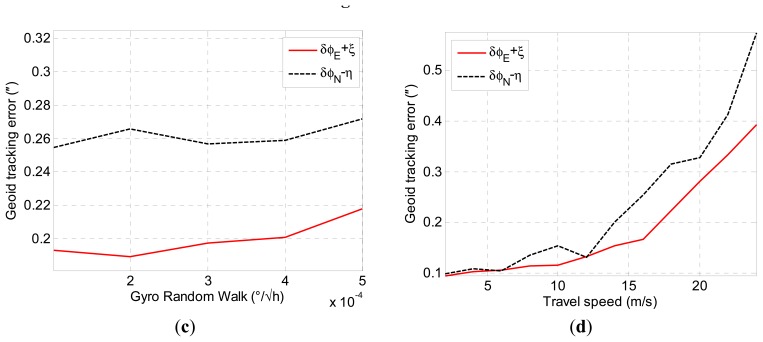
(**a**) Geoid tracking error with different GNSS accuracy; (**b**) the geoid tracking error with respect to the accelerometer noise; (**c**) the geoid tracking error with respect to the gyro noise; (**d**) The geoid tracking error with respect to travel speed.

We first investigate the ability of the tracking geoid for INS/GNSS integration with different accuracies for the accelerometer, gyroscope and GNSS. Figure 8a depicts the INS/GNSS geoid tracking error with different GNSS velocity accuracies (the GNSS position accuracy is of the same scale with velocity accuracy) for the given inertial sensors accuracy in Table 1. It can be seen that the geoid tracking error is reduced as the GNSS accuracy improves. The white-noise error of the accelerometer is another primary factor for INS/GNSS integration. Figure 8b shows the geoid tracking accuracy with respect to the accelerometer noise. The simulation results shows that high-precision GNSS and low-noise accelerometer are required to obtain the DOV data with 1″ accuracy. In contrast, the gyroscope noise has little influence on the geoid tracking error, as can be seen in Figure 8c.

The INS/GNSS integration is implemented by an RTS smoother based on the Kalman filter. It takes some time for the state estimation of the Kalman filter to converge to the true state when the DOV changes with positions. When the vehicle travels at a high speed, the filter has difficulty responding to the change of DOV, and the estimation error increases accordingly. Figure 8d shows the geoid tracking error of INS/GNSS integration with respect to the travel speed. It can be seen that the geoid tracking error increases with the travel speed of the vehicle. It should be noted that the proposed method can be only applied in a land vehicle or ship due to the limitation of travel speed.

### 4.2. Analysis of Attitude Reference Error

In this paper, a star sensor, which can measure the attitude with high precision, serves as an attitude reference to calculate the INS/GNSS attitude error, and the LGU is used to maintain the attitude reference when the star sensor has inaccuracies in the dynamic situation. The large standard deviations of DOV estimation errors are caused mostly by the LGU attitude errors, as shown in Table 2. Therefore, we will discuss the LGU errors induced by the attitude initialization error, the bias and angular random walk (ARW) error of gyroscopes and investigate the characteristics of these error sources by simulations.

Despite the high precision of the initializing attitude, the LGU attitude errors are drifting during traveling. The dynamics of LGU attitude error can be described by Equation (7) in Section 2. Considering the initial error of the LGU attitude only and ignoring $\omega_{en}^{n}$, which is a minor term when compared with $\omega_{ie}^{n}$, Equation (7) can be rewritten as:

(31) $$\dot{\Phi }=-\omega_{ie}^{n}\times \Phi$$

**Figure 9.**
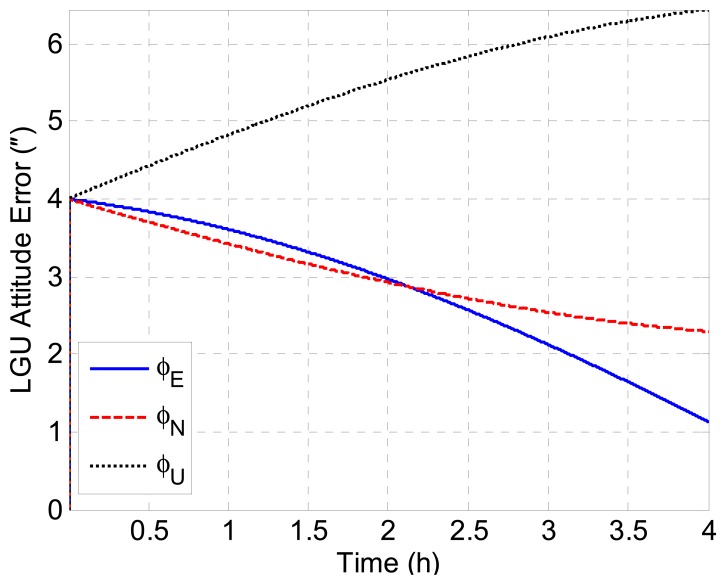
LGU attitude error introduced by initialization error.

We can see that the attitude errors have an inherent frequency *ω_ie_*. Figure 9 illustrates the growth of LGU attitude errors introduced by the initial alignment error when the gyroscopes are error-free. As can be seen, the attitude errors behave as trends during a segment of 4 h.

**Figure 10.**
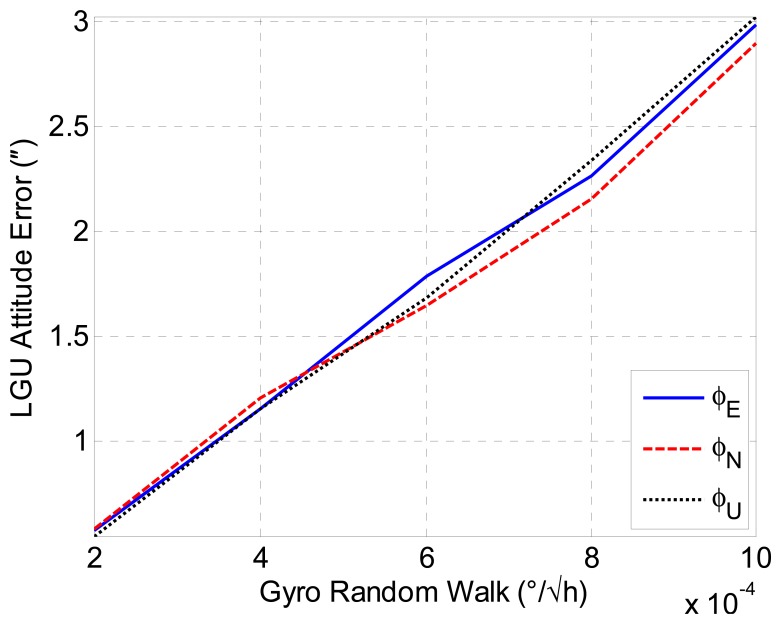
LGU attitude error with respect to gyro angular random walk (ARW) accuracy.

Then, we will discuss the gyro-induced attitude errors of the LGU. As the gyro biases are estimated and compensated for in the INS/GNSS integration, the ARWs are the major errors of the gyroscopes. The attitude accuracy of the LGU under different standard deviations of the gyroscopes ARW errors is investigated by simulations. The simulation time is four hours. The standard deviations of the attitude errors are used to evaluate the performances of attitude accuracy for each simulation trajectory. Figure 10 shows the mean value of 100 Monte Carlo simulations for LGU attitude error relative to gyro ARW accuracy. It is noted that a gyro ARW of 0.0002 °/√h is required for the DOV measurement with 0.5″ accuracy, and thus, it is extremely critical for the inertial sensor.

A random walk process can be described by a differential equation below:

(32) $$x_{k}-x_{k-1}=w_{k}$$

where *w* is a Gaussian white-noise process. The transfer function of the random process can be calculated by *z*-transform:

(33) $$H(z)=\frac{1}{1-z^{-1}}$$

The power spectral density (PSD) is given by:

(34) $$S(\omega )=\sigma^{2}{\left|H(e^{j\omega })\right|}^{2}=\frac{\sigma^{2}}{2-2\cos\omega }$$

where *σ*^2^ is the variance of the white-noise process.

**Figure 11.**
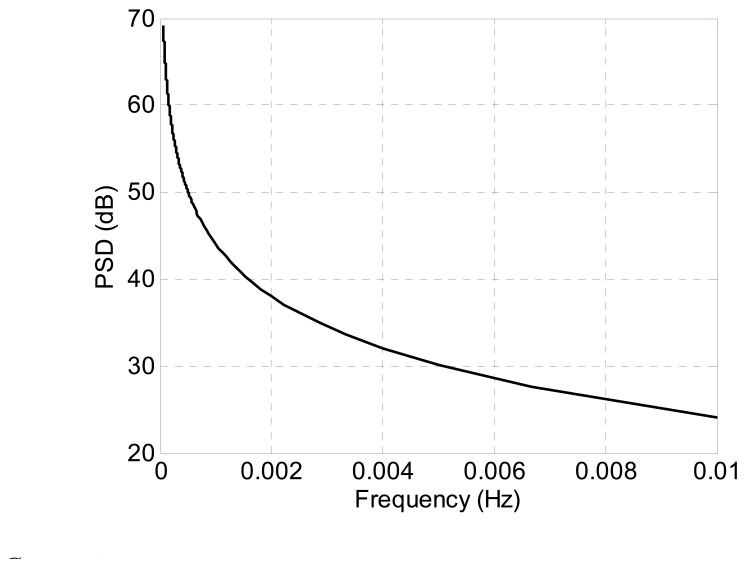
Power spectral density (PSD) of the angular random walk.

As shown in Figure 11, the PSD of ARW has a low-pass characteristic, and the power of the signal is concentrated in the relative low-frequency domain. Consequently, the LGU attitude error introduced by the gyroscopes' ARWs exhibits a characteristic of trend which is similar to the initialization-induced error.

### 4.3. Trend Error Correction

**Figure 12.**
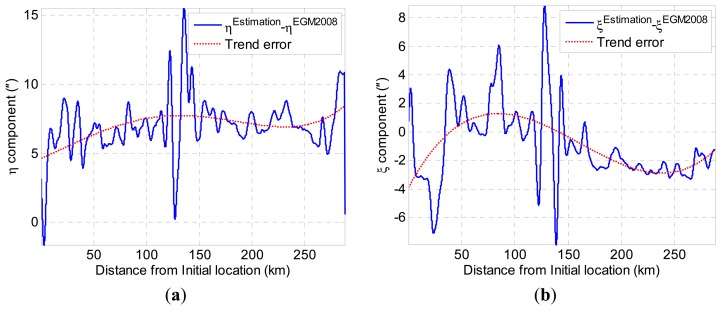
(**a**) Trend error extraction for *η*; (**b**) trend error extraction for *ξ*.

**Figure 13.**
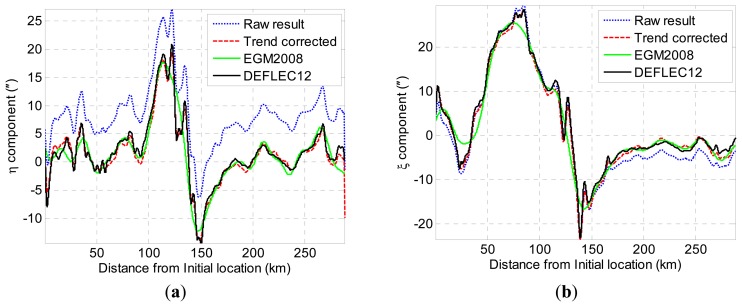
(**a**) Trend error correction for *η*; (**b**) trend error correction for *ξ*.

It is well known that the EGM2008 is the most accurate global gravity model published, which can provide accurate long-wavelength (which is longer than 10 km) components of gravity disturbances [25]. Although this long-wavelength information cannot reflect the details of the gravity disturbances, they can be utilized to remove the trend error in the gravimetry measurement data [26]. The proposed method attempts to utilize the EGM2008 to remove the trend error in DOV estimation. There are three steps to remove the trends error. First, the long-wavelength components of DOV are removed by subtracting the EGM2008 DOV data from raw DOV estimation data; thus, the residual of DOV contains only short-wavelength components. Second, the trend errors of the DOV residual are fit by a low-order polynomial. Figure 12a,b shows the trend errors extracted from the difference between the raw DOV estimation data and EGM2008 data. The short-wavelength components are extracted when the trends are removed. Third, the ultimate results of DOV can be obtained by combining the short-wavelength components and the EGM2008 gravity data. The final DOV results are shown in Figure 13a,b. As is expected, the trend errors are mostly removed, and the ultimate result contains more detailed information than the EGM2008 gravity model.

Table 3 shows the DOV error of the EGM2008, in which it is compared with the DEFLEC12 model along the trajectory. According to Table 3, we can see that the standard deviation (SD) of the EGM2008 vertical deflection errors is approximately 2″. Monte Carlo simulations are implemented to verify the feasibility of the trends correction method. The statistical analysis of DOV measurements in the simulations is listed in the following tables. It can be seen from Table 4 that the average errors of the preliminary estimation for *ξ* and *η* components (before trends correction is applied) with 100 Monte Carlo simulations are 1.50″ and 1.23″, respectively, and the maximum errors are 3.32″ and 2.35″. When the trends' correction algorithm is applied, the average errors of the *ξ* and *η*; components are reduced to 0.69″ and 0.85″, and the maximum errors are reduced to 0.85″ and 1.03″, as Table 5 shows.

**Figure 14.**
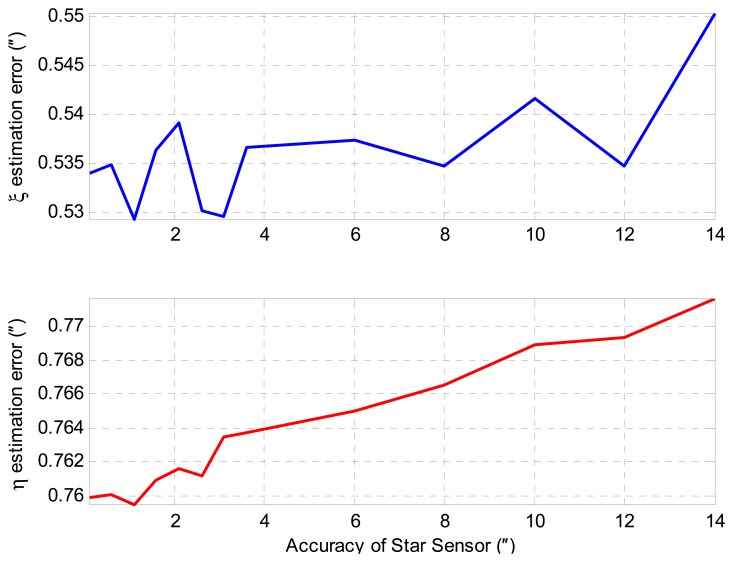
The effect of star sensor accuracy on DOV estimation results after trend error correction.

According to the simulation results, we can see that that the trends' correction algorithm can effectively reduce the trend errors, which are mainly caused by the initial error and the ARW-induced error of attitude reference. In this sense, the precision of the initial attitude has little influence on the DOV measurement results. As can be seen from Figure 14, the increments of DOV estimation errors are less than 0.02″ when the accuracy of star sensor changes from 0.1″ to 14″.

## 5. Discussion and Conclusion

A new method for the dynamic measurement of DOV is proposed in this paper based on an INS/GNSS integration system and a star sensor. The feasibility of this method is investigated through extensive simulations and detailed error analysis. The preliminary results show that considerable systemic errors are contained in DOV components. The LGU attitude error, which is induced by the initializing error and gyros error, is the primary error source in the measurement. Fortunately, the systemic errors behave as the characteristics of trends, while the EGM2008 gravity model is considered to be accurate in the long-wavelength domain. Thus, an algorithm for trend error correction is developed with the aid of the EGM2008 gravity model. The estimation results of the *ξ* and *η*components are significantly improved from 1.50″ and 1.23″ to 0.69″ and 0.85″, respectively, when the trends error correction algorithm is applied.

The new method has three distinct features compared to the traditional inertial vector gravimetry. First, DOVs are calculated by using the transformation matrix between the geodetic and astronomical coordinates, rather than the difference between the GNSS-derived accelerations and INS-sensed specific force. Therefore, the calculation of the kinetic acceleration is avoided. As the velocity estimation technique using differences of carrier-phase measurements can reach an accuracy at the mm/s level [27] and the Precise Point Position (PPP) technique enables the GNSS to position with 0.1-m accuracy, a stand-alone GNSS receiver may meet the demands of the proposed method. Second, a single-axis rotation inertial navigation system is adopted in the new method. Thus, the observability of the system errors is improved, and the bias errors of the inertial sensors are restrained by the tuning structure. Third, trend errors can be removed with the survey data of a single track in the new method. As was mentioned in Section 1, repeated tracks of survey data are required in traditional vector gravimetry for the WCF technique to remove the systemic errors. This significantly increases the cost and time for gravity survey. In contrast, in the new method the trend errors are removed and directly compensated for by the EGM2008 global gravity model in a single survey line.

The simulation result shows that the proposed method is limited by the speed of the vehicle, because the geoid tracking errors of INS/GNSS integration will increase when the vehicle travels at a high speed. Therefore, this method is not suitable for the airborne application. A ship or land vehicle with a speed lower than 25 m/s is recommended as the carrier for the measurement.

As the trend errors caused by the initial error of LGU can be effectively removed by the trend error correction algorithm, the precision of the star sensor, which is utilized to initialize the attitude of LGU, has little influence on the DOV estimation results. Thus, we can improve the proposed method by using the attitude output of INS/GNSS integration to initialize LGU; then, there is no requirement of a star sensor at all. This improvement can considerably reduce the cost and improve the efficiency for DOV measurement. Moreover, the survey can be carried out in the daytime and has no limitation on weather conditions.

It should be noted that the performance of the trend error correction depends on the accuracy of global gravity model in the long-wavelength domain. An improved gravity model, such as global gravity model plus (GGM plus) [28], may yield more advanced results. As new satellite gravimetry missions (e.g., Gravity and Ocean Circulation Explorer (GOCE)) can provide high precision gravity information, a more accurate geoid reference will be obtained. It is expected that the newly-developed global gravity models will be more accurate and have higher resolution when the new satellite gravimetry data is available. Accordingly, the accuracy of the proposed method will be further improved with the aide of these new global gravity models.

## Figures and Tables

**Table 1.** The performances of sensors for simulation.

**Sensors**	**Characteristics**	**Magnitude (1σ)**
Gyroscope	Constant bias	0.003 °/h
	White noise	0.0004 °/√h

Accelerometer	Constant bias	2e−4 m/s^2^
	White noise	1e−5 m/s^2^

GNSS velocity	Horizontal error	0.003 m/s
	Height error	0.003 m/s

GNSS position	Horizontal error	0.2 m
	Height error	0.5 m

Star sensor	Attitude error	3″

**Table 2.** Statistical analysis of the simulation result (″).

	**Standard Deviation**	**Mean**
$\delta \phi_{E}^{\text{INS}}+\xi^{\text{DEFLEC}12}$	0.30	0.00
$\delta \phi_{N}^{\text{INS}}-\eta^{\text{DEFLEC}12}$	0.45	0.01
$\delta \phi_{E}^{\text{LGU}}$	2.62	3.11
$\delta \phi_{N}^{\text{LGU}}$	1.51	1.57
*ξ^Estimation^* − *ξ^DEFLEC^*^12^	2.63	−3.11
*η^Estimation^* − *η^DEFLEC^*^12^	1.59	−1.56

**Table 3.** DOV error of EGM2008 (″).

	**SD**	**Mean**
*ξ^EGM2008^* − *ξ^DEFLEC^*^12^	2.26	−0.76
*η^EGM2008^* − *η^DEFLEC^*^12^	2.00	−0.11

**Table 4.** The statistical analysis of preliminary DOV estimation result (″).

	**Average Error**	**Maximum Error**
*ξ^Estimation^* − *ξ^DEFLEC^*^12^	1.50	3.32
*η^Estimation^* − *η^DEFLEC^*^12^	1.23	2.35

**Table 5.** The statistical analysis of ultimate DOV estimation result (″).

	**Average Error**	**Maximum Error**
*ξ^Estimation^* − *ξ^DEFLEC^*^12^	0.69	0.85
*η^Estimation^* − *η^DEFLEC^*^12^	0.84	1.03
